# News and Coming Events

**Published:** 1908-08-08

**Authors:** 


### News and Coming Events.

Among the charitable bequests under the will of the late Mrs. Hannah Bland are sums of £500 each to the Samaritan Hospital for Women, the Middlesex Hospital, and the Marylebone General Dispensary.

The Treasurer of the London Homœopathic Hospital has received £1,000 from the estate of the late Mrs. George Fielder to endow a bed in the new extension.

It has been announced in Cardiff that Mrs. John Nixon, of Queen Anne's Gate, London, has promised 10,000 guineas for the endowment of a children's ward in the Cardiff Infirmary.

In the fifteenth list of units of the Territorial Force which have received the recognition of the Army Council the following units of the R.A.M.C. (T.) are included: 2nd Scottish General Hospital, 2nd London Sanitary Company, 5th Southern General Hospital.

London Homœopathic Hospital.—Lord Cawdor, the Treasurer of the London Homœopathic Hospital, Great Ormond Street, W.C., has received a cheque for £2,000, being the amount of a legacy bequeathed to the hospital by the late Mr. Caleb Ashworth Tate.

King Edward's Hospital Fund for London.—The Hon. Secretaries of King Edward's Hospital Fund for London have received at the Bank of England the eleventh annual grant of £1,000 from the trustees of the London Parochial Charities, to be applied to the maintenance of convalescent hospitals.

The half-yearly Court of Governors of the East London Hospital for Children was held at Shadwell on July 27. Not only is the hospital free from debt, but £3,000 has been added to permanent investments. Improvements urgently required for the Convalescent Home cannot, however, be carried out at present owing to lack of funds for that purpose.

The following will be the lecturers at the Royal College of Physicians for 1909: Goulstonian, Dr. A. E. Russell; Milroy, Dr. R. T. Hewlett; FitzPatrick, Sir T. Clifford Allbutt; Oliver-Sharpey, Dr. C. S. Sherrington; Croonian, Dr. Lazarus Barlow (1909), Dr. A. Gamgee (1910). The Harveian oration on October 19 will be by Dr. J. A. Ormerod, and in November Dr. L. Guthrie will deliver the FitzPatrick, and Dr. Dudgeon the Horace Dobell lectures.

At the Interim Court of Governors of the Brompton Hospital for Consumption and Diseases of the Chest, held on July 24, the Right Hon. the Earl of Derby, P.C., K.C.V.O., C.B., was elected President of the hospital in succession to his father, the late Earl. It was announced that a certain number of beds have now been provided at the Frimley Sanatorium for paying patients at 25s. per week, some of which are already occupied. Also that the working details of a scheme for voluntary notification have now been completed and will come into operation after the summer vacation. Practically the whole of the medical officers of health of the London boroughs have expressed their warm approval of the scheme. Donations received since the last Court included £500 from the Trustees of Smith's Charity and £100 from the City of London Corporation.

Sir William J. Collins, M.S. (Lond.), F.R.C.S. (Eng.), has been appointed senior ophthalmic surgeon to the Hampstead Hospital (with which is now amalgamated the North-West London Hospital).

At the Chesterfield Examination in Dermatology recently held at St. John's Hospital for Diseases of the Skin, Leicester Square, the Chesterfield Silver Medal was awarded to Frank E. Tylecote, M.D., M.R.C.P., D.P.H., Resident Medical Officer of Barnes Convalescent Hospital, Cheadle, Manchester.

The death is announced, on July 30, of Dr. T. Orme Dudfield, Medical Officer of Health for the Royal Borough of Kensington, at the age of seventy-five years. Dr. Dudfield was a St. George's man; he became a member of the Royal College of Surgeons in 1861, and a doctor of medicine of St. Andrew's in the same year. He had been medical officer of health for Kensington for 37 years, and was a Past-President of the Society of Medical Officers of Health.

On August 1 the Home Secretary, replying to a question by Mr. Bramsdon, said he had seen the newspaper reports of three recent deaths under anæsthetics in London hospitals. He is in communication with the Lord President of the Council, and through him with the General Medical Council, on the question whether a course of instruction in the administration of anæsthetics can be included in all cases in the course of study required for a medical qualification, and he thinks a formal inquiry by Royal Commission or Committee may with advantage be postponed, at any rate until he knows what action the medical authorities are prepared to take in this matter.

At the quarterly *comitia* of the Royal College of Physicians held on July 30, Sir Richard Douglas Powell presiding, the following officers were elected: Censors, Norman Moore, M.D., F. de H. Hall, M.D., S. J. Sharkey, M.D., and J. K. Fowler, M.D.; Treasurer, Sir Dyce Duckworth, M.D.; Emeritus Registrar, Sir H. A. Pitman, M.D.; Registrar, E. Liveing, M.D.; Harveian Librarian, J. F. Payne, M.D.; Assistant Registrar, J. A. Ormerod, M.D.; Library Committee, W. Osler, M.D., H. D. Rolleston, M.D., A. F. Voelcker, M.D., C. A. Mercier, M.D.; Curators of the Museum, W. Cayley, M.D., Sir W. H. Allchin, M.D., S. J. Sharkey, M.D., W. Hunter, M.D.

At the same time the following were elected examiners in the subjects indicated for the ensuing year: First Examination, W. H. Hurtley, D.Sc., G. Senter, Ph.D., B.Sc., D. F. D. Turner, M.D., A. W. Porter, B.Sc.; materia medica and pharmacy—C. Ogle, M.D., J. J. Perkins, M.D., A. P. Beddard, M.D., R. A. Young, M.D., O. F. F. Grünbaum, M.D. Second Examination.—Physiology—W. D. Halliburton, M.D., M. S. Pembrey, M.D.; Anatomy—P. Thompson, M.D. Final Examination.—Medicine—J. A. Ormerod, M.D., S. H. C. Martin, M.D., T. D. Acland, M.D., H. D. Rolleston, M.D., Sir Edwin Perry, M.D., Sir Hugh R. Beevor, M.D., J. Galloway, M.D., W. E. Wynter, M.D., W. J. Hadley, M.D., L. Humphry, M.D.; Midwifery—J. Phillips, M.D., H. R. Spencer, M.D., W. J. Gow, M.D., T. W. Eden, M.D., G. H. D. Robinson, M.D.; Public Health—W. H. Wilcox, M.D., A. G. R. Foulerton, F.R.C.S.

The Council of Sheffield University, at its meeting on July 31, appointed Dr. Ralph P. Williams, M.D., B.S. (Lond.), D.P.H. (Oxf.), to be Professor of Public Health.

A society of members of the medical profession has been formed in Tokio with the object of studying means of stamping out beri-beri, which attacked many soldiers during the recent war in Manchuria. The chief of the Japanese Army Medical Department has accepted the presidency of the society.

Dr. Seaton, the medical officer of health for the administrative county of Surrey, in his report for the year 1907, just published, devotes a section to the working of the Midwives Act in the county. During the year there were 190 certified midwives practising in the county, and of these the great majority are described as being cleanly in their habits and in their dwellings. They attended 4,798 women in child-birth, and there were ninety-two still-births among this number. During the ten years 1895-1904 the number of cases of puerperal fever notified was 278, or an average of 27.8 per annum; and during the three following years, 1905-1907, when the Act was in operation, the number was sixty-six, or an average of twenty-two per annum. Hence, although the number of births in the county has increased (although not in proportion to the population), the number of puerperal fever cases has been reduced by nearly 20 per cent.; while infantile ophthalmia is said to have been "of very rare occurrence." There are still about seventy uncertified midwives practising in the county, who will be practically legalised until April 1, 1910, after which date they will be suppressed. The report declares that the replacement of these persons by younger women, practised in midwifery as now taught, would mean a diminished child-birth death-rate and a diminished infant mortality; but it is at present by no means clear how the future wants of the community are to be supplied. In many districts it cannot possibly pay a competent midwife to establish herself unless she is subsidised by a national or county association.

The new out-patients' department of the Hospital for Sick Children, Great Ormond Street, was opened without ceremony on August 4. It has been built by means of a fund of £50,000 placed in the hands of trustees by Mr. W. W. Astor for the improvement of the hospital. The new building adjoins the main block and occupies the site of the old Working Men's College.

THE HOSPITAL August 8, 1908.

